# Supplementary material for: Smooth Interpolating Curves with Local Control and Monotone Alternating Curvature
Source: Comput Graph Forum. 2022 Oct 6;41(5):25–38. doi: 10.1111/cgf.14600 (PMC9827861; doi:10.1111/cgf.14600)
Supplement: Supplementary file 1 — Supplement Material [file CGF-41-25-s001.zip › Local-Smooth-Interpolating-MonoCurvature/extern/clothoids/submodules/Utils/docs/api-cpp/program_listing_file_Utils_TicToc.hxx.html]

Program Listing for File TicToc.hxx — Utils v1.2

### Navigation

- index
- toc
- Utils »
- Program Listing for File TicToc.hxx

# Program Listing for File TicToc.hxx¶

↰ Return to documentation for file (`Utils/TicToc.hxx`)

```
/*--------------------------------------------------------------------------*\
 |                                                                          |
 |  Copyright (C) 2017                                                      |
 |                                                                          |
 |         , __                 , __                                        |
 |        /|/  \               /|/  \                                       |
 |         | __/ _   ,_         | __/ _   ,_                                |
 |         |   \|/  /  |  |   | |   \|/  /  |  |   |                        |
 |         |(__/|__/   |_/ \_/|/|(__/|__/   |_/ \_/|/                       |
 |                           /|                   /|                        |
 |                           \|                   \|                        |
 |                                                                          |
 |      Enrico Bertolazzi                                                   |
 |      Dipartimento di Ingegneria Industriale                              |
 |      Universita` degli Studi di Trento                                   |
 |      Via Sommarive 9, I-38123 Povo, Trento, Italy                        |
 |      email: enrico.bertolazzi@unitn.it                                   |
 |                                                                          |
\*--------------------------------------------------------------------------*/


#pragma once

#ifndef DOXYGEN_SHOULD_SKIP_THIS
#ifndef UTILS_TIC_TOC_dot_HH
#define UTILS_TIC_TOC_dot_HH

#ifndef UTILS_OS_WINDOWS
  #include <chrono>
  #include <thread>
#endif
#endif

namespace Utils {

#ifdef UTILS_OS_WINDOWS
  class TicToc {

    typedef double real_type;
    int64_t   m_frequency;   // ticks per second
    int64_t   m_t1, m_t2;    // ticks
    real_type m_elapsed_time;

    TicToc( TicToc const & ) = delete;
    TicToc const & operator = ( TicToc const & ) const = delete;

  public:

    TicToc();
    ~TicToc() {}

    void tic();

    void toc();

    real_type elapsed_s()  const { return 1e-3*m_elapsed_time; }

    real_type elapsed_ms() const { return m_elapsed_time; }

  };

  void sleep_for_seconds( unsigned s );
  void sleep_for_milliseconds( unsigned ms );

#else

  class TicToc {

    typedef double real_type;
    #ifdef TIC_TOC_USE_HRC
    typedef std::chrono::high_resolution_clock clock;
    #else
    typedef std::chrono::steady_clock clock;
    #endif

    using elapsed_resolution = std::chrono::microseconds;

    clock::time_point m_start_time;
    clock::time_point m_stop_time;

    elapsed_resolution m_elapsed_time;

    TicToc( TicToc const & ) = delete;
    TicToc const & operator = ( TicToc const & ) const = delete;

   public:

    TicToc()
    : m_elapsed_time(0)
    { this->tic(); }

    ~TicToc() {}

    void tic();

    void toc();

    real_type elapsed_s() const;

    real_type elapsed_ms() const;
  };

  inline
  void
  sleep_for_seconds( unsigned s ) {
    std::this_thread::sleep_for(std::chrono::seconds(s));
  }

  inline
  void
  sleep_for_milliseconds( unsigned ms ) {
    std::this_thread::sleep_for(std::chrono::milliseconds(ms));
  }

#endif

}

#endif
```

### Quick search

### Table of Contents

- rang - Colors for your Terminal
- Zstr
- C++ API

«
hide menu

menu
sidebar
»

### Navigation

- index
- toc
- Utils »
- Program Listing for File TicToc.hxx

© Copyright 2021, Enrico Bertolazzi.
Created using Sphinx 4.2.0.
